# Magnetic Resonance Imaging Derived Biomarkers of IDH Mutation Status and Overall Survival in Grade III Astrocytomas

**DOI:** 10.3390/diagnostics10040247

**Published:** 2020-04-23

**Authors:** Paola Feraco, Antonella Bacci, Patrizia Ferrazza, Luc van den Hauwe, Riccardo Pertile, Salvatore Girlando, Mattia Barbareschi, Cesare Gagliardo, Alessio Giuseppe Morganti, Benedetto Petralia

**Affiliations:** 1 Neuroradiology Unit, S. Chiara Hospital, Trento, Largo Medaglie d’oro 9, 38122 Trento, Italy; benedetto.petralia@apss.tn.it; 2 Department of Experimental, Diagnostic and Specialty Medicine (DIMES), University of Bologna, Via San Giacomo 14, 40122 Bologna, Italy; alessio.morganti2@unibo.it; 3 Department of Neuroradiology, Ospedale Bellaria, IRCSS Istituto delle Scienze Neurologiche, Via Altura, 9 40100 Bologna, Italy; antonella.bacci@isnb.it; 4 Radiation Oncology Unit, APSS, Largo Medaglie d’oro 9, 38122 Trento, Italy; patrizia.ferrazza@apss.tn.it; 5 Department of Radiology, University of Antwerp, Wilrijkstraat 10, B2650 Edegem (Antwerp), Belgium; lucvdhauwe@mac.com; 6 Clinical and Evaluative Epidemiology Department-Trento Health Service, Trento, Via de Gasperi 79, 38100 Trento, Italy; riccardo.pertile@apss.tn.it; 7 Pathology Unit, S. Chiara Hospital, Largo Medaglie d’oro 9, 38122 Trento, Italy; salvatore.girlando@apss.tn.it (S.G.); mattia.barbareschi@apss.tn.it (M.B.); 8 Section of Radiological Sciences, Department of Biomedicine, Neuroscience and Advanced Diagnostics, University of Palermo, Via Del Vespro 129, 90127 Palermo, Italy; cesare.gagliardo@unipa.it

**Keywords:** isocitrate dehydrogenase (IDH) mutation, diffusion weighted imaging (DWI), apparent diffusion coefficient (ADC), anaplastic astrocytomas, overall survival, radiogenomics

## Abstract

The evaluation of the isocitrate dehydrogenase (IDH) mutation status in the glioma decision-making process has diagnostic, prognostic and therapeutic implications. The aim of this study was to evaluate whether conventional magnetic resonance imaging (MRI) and apparent diffusion coefficient (ADC) can noninvasively predict the most common IDH mutational status (R132H) in GIII-astrocytomas and the overall survival (OS). Hence, twenty-two patients (9-F, 13-M) with a histological diagnosis of GIII-astrocytoma and evaluation of IDH-mutation status (12-wild type, 10-mutant) were retrospectively evaluated. Imaging studies were reviewed for the morphological feature and mean ADC values (ADCm). Statistics included a Fisher’s exact test, Student’s *t*-test, Spearman’s Test and receiver operating characteristic analysis. A *p* ≤ 0.05 value was considered statistically significant for all the tests. A younger age and a frontal location were more likely related to mutational status. IDH-wild type (Wt) exhibited a slight enhancement (*p* = 0.039). The ADCm values in IDH-mutant (Mut) patients were higher than those of IDH-Wt patients (*p* < 0.0004). The value of ADC ≥ 0.99 × 10^−3^ mm^2^/s emerged as a “cut-off” to differentiate the mutation state. In the overall group, a positive relationship between the ADCm values and OS was detected (*p* = 0.003; r = 0.62). Adding quantitative measures of ADC values to conventional MR imaging could be used routinely as a noninvasive marker of specific molecular patterns.

## 1. Introduction

In the 2016 World Health Organization (WHO) classification of Tumors of the Central Nervous System [1], molecular parameters were integrated with histopathology into glioma characterizations to create more biologically homogenous groups. Hence, grades II, III and IV gliomas are divided into isocitrate dehydrogenase (IDH) mutant (Mut) glioma and IDH wild-type (Wt) glioma. An IDH mutation is a distinctive genomic alteration that plays important roles in gliomagenesis and occurs in 70% to 90% of grade II/III gliomas. Mutations of the IDH gene family produce oncometabolite 2- hydroxyglutarate (2-HG), leading to a slower growth of tumor cells compared to the wild types [2]. The detections IDH statuses have significant therapeutic and prognostic implications in terms of prolonged survival and chemosensitivity in the IDH-Wt subgroup [3,4,5,6].

The analysis of histopathological specimens through immunohistochemistry and genomic sequence is the gold standard method for detecting IDH mutations in patients with glioma. However, these methods are invasive, and, due to intratumoral heterogeneity, standard biopsies may lead to an incorrect result. Moreover, some tumors can be not resectable, since they are located in a critical eloquent area. On the other hand, medical imaging procedures can evaluate the entire tumor in a noninvasive and reproducible way.

A MRI is the primary noninvasive modality of choice for the early diagnostic work-up of gliomas. Considering that IDH gene mutations may reflect alterations in metabolism, cellularity and angiogenesis, which may manifest characteristic features on an MRI [7,8,9], the identification of specific MRI biomarkers could be of great interest for the management of patients with brain gliomas. Various MRI advanced techniques, such as diffusion weighted imaging (DWI), perfusion weighted imaging (PWI) and proton magnetic resonance spectroscopy (^1^H-MRS), were also applied to detect the IDH mutation status [10]. In particular, ^1^H-MRS can directly detect the presence of 2-HG and the consequent IDH mutational status. On the other hand, the use of this technique is usually limited to the academic world.

A different approach may rely on DWI usage that can provide, in a noninvasive way, direct insight into the microscopic physical properties of tissues. Indeed, DWI, evaluating the Brownian movement of water molecules, indirectly reflects cellularity within the lesions by means of apparent diffusion coefficient (ADC) values [11]. ADC is a measure of the magnitude of the random motion of water molecules within a tissue, and it is calculated by using data from DWI pulse sequences. It has been widely described that ADC maps are useful to provide information about tissue microarchitecture. In particular, inverse significant correlations between ADC, cell count and ki67 have been reported [12,13]. Recently, some studies have shown that ADC maps may easily discriminate the IDH mutations status, but most of them did not consider a homogeneous group of gliomas in terms of grading and histology [14,15,16,17,18]. Indeed, ADC can be influenced by histology and grading [19,20,21,22]. Therefore, we decided to focus our study, according the 2016 WHO brain tumor classification, on grade III astrocytic tumors only. Despite that grade III astrocytomas have always been considered to be high-grade tumors and treated as glioblastomas (GBMs), the mutate IDH group was reported to show overall survival (OS) comparable to corresponding grade II tumors [23]. This finding is relevant, because this group could be clinically managed as low-grade tumors. Thus, the aim of our study was to preoperatively predict the IDH mutation status in grade III astrocytoma using conventional MRI and ADC maps. Moreover, we wanted to define if ADC could be an MRI-derived biomarker predictive of OS.

## 2. Materials and Methods

### 2.1. Patient Cohort

This retrospective study was approved by the Human Research Ethics Committee of the “Azienda Provinciale per i Servizi Sanitari (APSS)” of Trento (Prot—MOLIMA—07 Mar 2019). Written informed consent was obtained from all participants.

Two-hundred patients who underwent surgical resection or stereotactic biopsy at our institution from July 2013 through October 2019 were selected. The inclusion criteria were: (1) definite histopathologic diagnosis of grade III astrocytoma based on the WHO 2016 classification criteria and (2) cMRI and DWI performed before treatment in the same MR session. Patients were excluded in cases of a lack of pre-surgical or pre-chemo/radiotherapy treatment imaging.

### 2.2. MR Imaging Acquisition

Images were acquired in the routine clinical work-up on a 1.5T MR imaging system (GE Optima MR450w 1.5T, Waukesha, WI, USA) with a 32-channel phased-array head coil. cMRI protocol was as follows: axial T1-weighted (T1w) fast spin echo (FSE) (TR 630 ms and TE 4.2 ms), axial T2-weighted (T2w) fast relaxation fast spin echo-propeller sequence (FRFSE-propeller) (TR 5300 ms and TE 96 ms; NEX 3.3) and axial T2w fluid-attenuated inversion recovery imaging (FLAIR) (TR/TE 11.000/92 ms and TI 2775 ms). Section thickness (5 mm), intersection gap (1mm) and FOV (240 × 240 mm) were uniform in all sequences. After IV contrast-agent injection (gadobutrol, 0.1 mmol/kg), a 3D fast-spoiled gradient-echo (FSPGR) sequence was acquired (TR 10 ms/TE 4.2 ms, 1mm isotropic voxel).

DWI was performed in the axial plane with an echo-planar sequence before injection of the contrast agent (gadobutrol, 0.1 mmol/kg). The imaging parameters used were as follows: TR/TE 7962/4.2 ms, NEX 1.0, section thickness 4 mm, intersection gap 1 mm and FOV 240 × 240 mm. The b-values were 0 and 1000 s/mm^2^ with diffusion gradients encoded in the x, y and z directions to generate 3 sets of diffusion-weighted images. ADC maps were automatically calculated by the integrated scanner software and converted into standard units (10^−3^ mm^2^/s).

All images were assessed by a European board-certified neuroradiologist, with 15 years of experience, blinded to the pathological diagnosis. MR imaging features included: (1) tumor location (frontal, parietal, temporal, insular or occipital); (2) hemisphere (right, left or bilateral); (3) signal intensity on T2w (homogeneous/heterogeneous); (4) well-defined borders on FLAIR images (yes/no); (5) peritumoral edema on FLAIR images (presence vs. absence) and (6) contrast enhancement presence (yes/no).

### 2.3. Pathology and Immunohistochemical Analysis

The nature and grade of the gliomas were determined according to the 2016 WHO classification [1]. IDH mutational status was determined by immunohistochemical antibody testing for the IDH1-R132H mutation, the most common glioma-derived mutation. Among IDH1-Mut, to exclude patients with concomitant 1p/19q co-deletion and, thus, a diagnosis of oligodendroglioma, fluorescence in-situ hybridization was performed. The methods for these molecular analyses have been described elsewhere [24,25].

### 2.4. Imaging Analysis

Images were analyzed on an off-line dedicated workstation (Advantage Workstation 4.3_8 GE) by using the provided analysis software (Functool, version 9.4.05a, GE Healthcare Systems).

**Figure 1:**
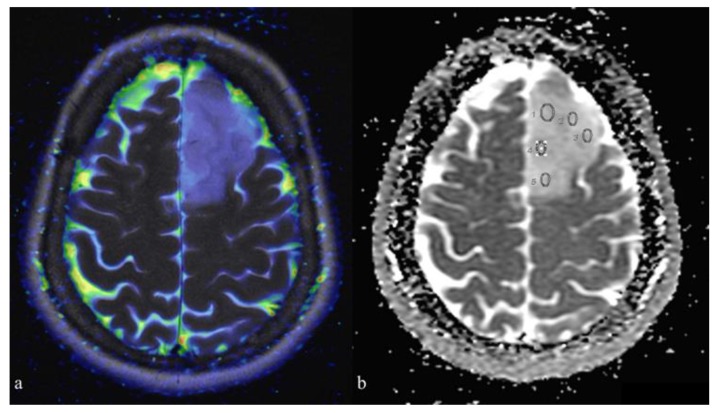
Placement of region on interests (ROIs) on the apparent diffusion coefficient (ADC) map: (**a**) co-registration between T2-weighted and color-coded ADC map (blue: restricted diffusion; red: facilitated diffusion); (**b**) placement of five elliptical ROIs on parametric ADC map within the tumor. Co-registration allows identification of the different components of the tumor and peritumoral region. Scale bar: 5 cm.

The placement of the region of interests (ROIs) was performed as previously described by Xing Z. et al. [15]. In particular, to ensure precise ROIs placements on the solid tumor components and avoid cystic, hemorrhagic and necrotic areas or peritumoral edema, the DWI images were co-registered with conventional MRI (FSE T1w pre-gadolinium and 3D FSPGR post-gadolinium and T2w FRFSE). Hence, the mean ADC values (ADCm) were measured by manually placing ROIs on the ADC maps (elliptical ROIs of approximately 15–30 mm^2^). From three to five non-overlapping ROIs were placed inside the tumor areas of the visually lowest ADC (Figure 1). Eventually, to minimize variances in ADCm values, relative ADC (rADCm) was obtained from the ratios of the tumor ADCm to the ADCm of a normal-appearing reference region (left cerebellum), defined on T2-weighted and contrast-enhanced T1-weighted images (CE-T1w). Moreover, for each patient, the lowest ADCm and rADCm values were chosen.

### 2.5. Statistical Analyses

Statistical analyses were performed using the SAS System (9.1.3 version). Descriptive statistics included mean, minimum and standard deviation of continuous variables and scores; in the case of categorical parameters, numbers and percentages distributions were used. The Student’s *t*-test and the Wilcoxon-Mann-Whitney test were used to test the differences of both ADCm and rADCm values between the IDH-Wt and IDH-Mut groups, controlling for the potential effects derived from tumor location and age. Fisher’s exact test was used to evaluate morphological characteristics’ prevalence in each IDH group. The receiver operating characteristic (ROC) was applied, and logistic regression modeling was performed to determine the ability of ADCm and rADCm to discriminate the IDH mutational status. The sensitivity, specificity, positive predictive value, negative predictive value and area under the curve based on optimum thresholds for variable parameters were calculated. OS in IDH-Mut and IDH-Wt groups were calculated with the Kaplan-Meier method. Comparison between survival curves was performed using the log-rank test. Spearman’s rho was calculated to assess the relationship between ADCm values and OS. A *p* ≤ 0.05 was considered statistically significant for all the tests.

## 3. Results

From the overall patient’s sample screened (*n* = 200), 120 tumors that underwent surgery were glioblastomas (GBMs). Among the grade III astrocytomas (*n* = 42), not all had both immunohistochemical and pretreatment MRI data available (*n* = 18). Moreover, not all had surgery in our hospital (*n* = 2). As a result, 22 patients (13 males and 9 females; mean age 52.3 ± 15.3 years; age range 32–82 years) were included in the study.

Demographic characteristics and conventional MRI features of the molecular subgroups are show in the Table 1.

Considering age, a significant difference was detected between the subgroups. The IDH-Mut patients were significantly younger than the IDH-Wt (*p* = 0.041), while no significant prevalence was found about sex. Tumors with IDH mutations were only located in the frontal (80%) and temporal lobes (20%), while there was no significant prevalence in location in the IDH-Mut groups.

**Figure 2:**
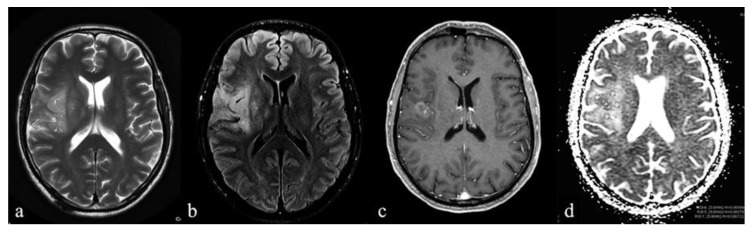
A 60-year-old man with an anaplastic astrocytoma IDH-Wt. (**a**,**b**) Axial T2-weighted and FLAIR images demonstrate a lesion with high signal intensity and indistinct borders on the right insular lobe; contrast-enhanced axial T1-weighted image (**c**) demonstrates a blurred contrast-enhancement within the lesion corresponding to the lower mean ADC (ADCm) value (**d**). Scale bar: 5 cm.

**Figure 3:**
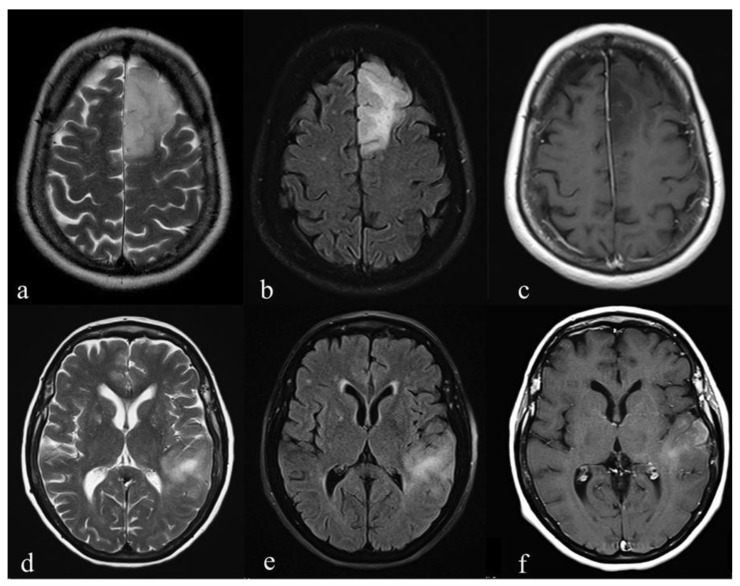
Pre-surgical conventional MR images of two patients with Grade III astrocytoma: (**a**–**c**) T2-weighted, fluid-attenuated inversion recovery images (FLAIR) and T1-weighted post-gadolinium images of an IDH-mutant (Mut) tumor show a lesion on the left frontal lobe. The borders are well-defined on T2-weighted (**a**) and FLAIR (**b**) images. No areas of T1-shortening are evident after IV contrast medium administration; (**d**–**f**) T2-weighted, FLAIR and T1-weighted post-gadolinium of an IDH-wild type (Wt) tumor show a hyperintense T2-weighted (**d**) and FLAIR (**e**) lesion on the left temporal lobe. Ill-defined borders (**d**,**e**) and a blurred contrast enhancement are detected (**f**). Scale bar: 5 cm.

There were no significant differences in the tumor borders and tumor homogeneity between the groups. IDH-Wt exhibited a slight enhancement (*p* = 0.039) compared with IDH-mut (Figure 2 and Figure 3).

**Figure 4:**
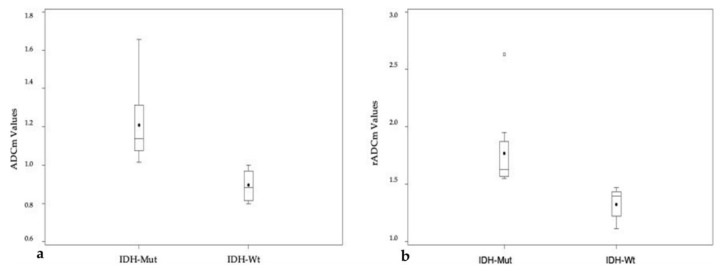
(**a**,**b**) Boxplots showing ADCm and relative ADC (rADCm) of IDH-Mut and IDH-Wt grade III astrocytomas. ADCm (**a**) and rADCm (**b**) differences are both significant for *p* < 0.01.

Significant differences between the ADCm and rADCm in IDH-Wt and IDH-Mut tumors were found (*p* = 0.0004 and *p* = 0.002, respectively), with higher values in IDH-Mut grade III astrocytomas compared to the IDH-Wt group (Figure 4 and Table 2). Higher ADCm values in IDH-Mut grade III astrocytomas were confirmed also after statistical correction for both location and age (*p* = 0.0012).

**Figure 5:**
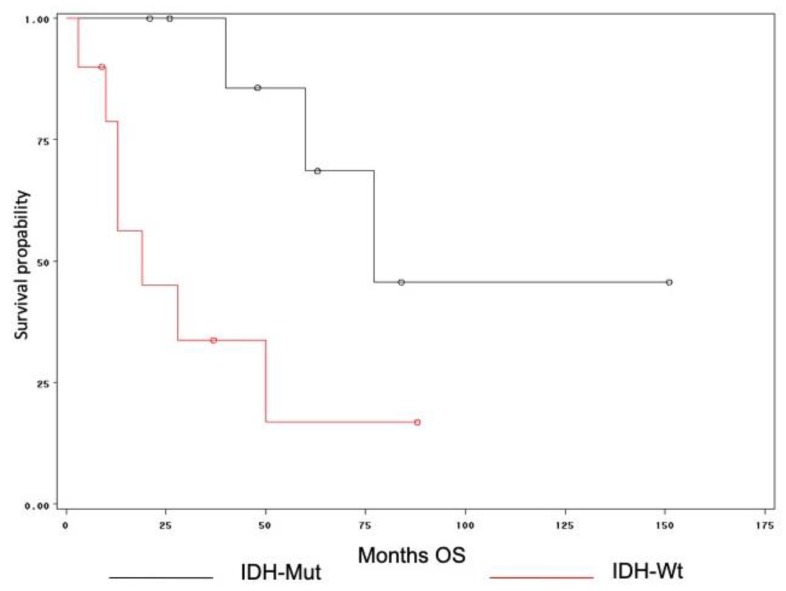
Kaplan-Meier curve with log-rank tests were used to examine differences in the overall survival (OS) among the different molecular subgroups. The red line represents the IDH-Wt tumors (*n* = 11), and the black line represents the IDH-Mut tumors (*n* = 9). The median OS in the IDH-Wt subgroup was significantly shorter (16 months) than the OS in the IDH-Mt (60 months; *p* < 0.001) subgroup.

**Figure 6:**
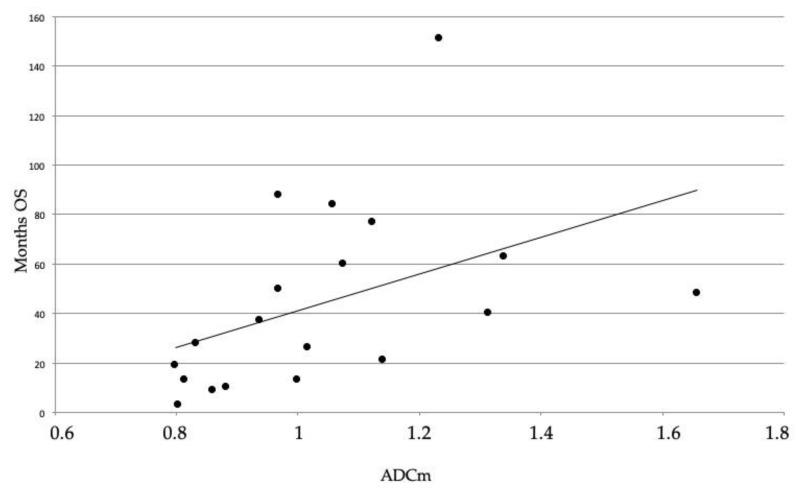
Spearman’s rho correlation between the ADCm values and overall survival of twenty patients showing a positive relationship between the variables (*p* = 0.003; r = 0.62), making the ADCm a biomarker predictor of the OS.

ROC analysis indicated that ADCm and rADCm variables had similar diagnostic performances in differentiating the IDH-Mut vs. IDH-Wt lesions. However, ADCm showed the highest area under the curve (AUC) value with a sensitivity and specificity of 100%. The value of ADC ≥ 0.99 *×* 10^−3^ mm^2^/s can emerge as a “cut-off” to differentiate the mutation state. Despite that the follow-up data of two patients (one IDH-Wt and one IDH-Mut) were not available, the difference in OS between the IDH-Wt and IDH–Mut subgroups was significant, with higher values for IDH-Mut (log-rank: chi-square 5.7156, DF 1, *p* < 0.016) (Figure 5). Finally, in the overall group, we found a positive relationship between ADCm values and OS (*p* = 0.003; r = 0.62) (Figure 6).

## 4. Discussion

In the current study, we explored whether conventional and diffusion MRI features could identify genetic subtypes of grade III astrocytoma. Our results showed significant differences between the groups. Considering the ages and morphological features of our population, consistent with previous studies, a younger age and a frontal location were more likely related to mutational status, while older ages and no location prevalence to wild-type status [26,27]. The current hypothesis is that the IDH-Mut tumors arise from a neural precursor population with a defined spatial and temporal location [4,5].

Our study showed a significant difference in contrast enhancement patterns between groups. Indeed, none of the IDH-Mut patients showed contrast enhancements, while five out of twelve IDH-Wt exhibited a slight enhancement (*p* = 0.039). Enhancement patterns in IDH-Wt could be related to the molecular similarity of this subgroup with GBMs, which show a more infiltrative behavior with blood barrier-disruption patterns on MRI. Furthermore, in our population, the presence/absence of edema was not related to a particular subgroup. Moreover, we did not find significant differences about the border’s features on FLAIR sequences, although the majority (60%) of IDH-Mut showed well-defined profiles, as reported in the literature [8,15].

However, ADCm was the unique parameter able to differentiate IDH-Mut from IDH-Wt tumors. Indeed, both the ADCm and rADCm values in the mutate group were significantly higher than those of the wild type. These results are consistent with the recent literature [10,20]. Indeed, after the 2016 WHO brain tumor classification update, some studies investigated the role of MRI in the prediction of the IDH mutational status in gliomas [15,16,17,18,19,20,21]. However, most of them evaluated the imaging characteristics of IDH molecular subgroups of mixed patient’s samples, from grade II to grade IV gliomas. Moreover, only a few grade III astrocytomas were reported to be included in these studies. The largest group of grade III astrocytomas was described by Xing Z. et al. [15], who reported characteristic IDH features of a heterogeneous group of grade II and grade III gliomas with 18 grade III astrocytomas (5 Mut and 13 Wt) but too small as a distinct group for a statistical analysis.

Since DWI has been mainly applied to assess tumor grade and cellularity [28,29], we decided to enroll only a defined histological grade so that cMRI features and ADC values were not dependent on the possible confounding effects of grading and histology. Indeed, the IDH gene mutation may reflects changes in metabolism, cellularity or angiogenesis [3]. In particular, voxels containing a smaller volume fraction of extracellular/extravascular water (high-cellularity tumor regions) present lower ADC values. On the other hand, those regions with a higher extracellular volume fraction (low-cellularity tumor regions) exhibit higher ADC values. Our results are in line with this evidence and support the hypothesis that there are significant differences between IDH-Mut and IDH-Wt tumors in terms of ultrastructural features that can be detected by ADC. Furthermore, our results identify a “cut-off” value of ADCm ≥ 0.99 × 10^−3^ mm^2^/s, to differentiate the mutational status with the sensitivity and specificity of 100%. Indeed, all IDH-Mut patients had ADCm values ≥ 0.99 × 10^−3^ mm^2^/s, with no overlaps with the IDH-Wt group.

Consistent with the literature, we detected a higher OS in IDH-Mut patients [30]. Moreover, in the overall group, the ADCm values were positively related to the OS (*p* = 0.003). Since IDH-Mut grade III gliomas have higher ADCm values, this parameter could also be considered as a good prognostic factor of the IDH mutation if compared with IDH-Wt.

Indeed, the ADC has been shown to correlate also with prognosis and treatment responses in patients with high-grade gliomas. In particular, a low pretreatment ADC value predicts a less favorable prognosis if compared with higher values [31,32].

Furthermore, although grade III astrocytomas have been considered to be high-grade tumors associated with poor OS, the mutate IDH group was reported to show an OS comparable to corresponding grade II tumors [33]. Hence, IDH-Mut grade III astrocytomas could be clinically managed as low-grade tumors. On the other hand, IDH–Wt tumors have been reported to exhibit GBM-like mutations and generally show very poor prognosis. Indeed, grade III IDH-Wt astrocytomas are most similar to GBMs and might represent misdiagnosed GBMs. Moreover, IDH-Wt astrocytomas exhibit a worse prognosis if compared with IDH-Mut GBMs [34]. Thus, the mutational status characterization of grade III astrocytomas might lead to new approaches for better overall patient management. In particular, for IDH-Mut grade III astrocytomas, a noninvasive method that provides an accurate presurgical diagnosis has the potential to improve patient treatment planning from the initial presentation. For example, the knowledge that a tumor is an IDH-Mut glioma would favor a more aggressive surgical resection, as recent studies suggest that a greater extent of resection independently correlates with survival in IDH-mutant astrocytic gliomas [35].

### Limits of the Study

Our study has some limitations that need to be acknowledged. First, it includes a small patient population, despite that the grading and histological profiles are homogeneous. Moreover, one could criticize the use of a 1.5T scanner, but it must be remembered that these scanners still represent the most popular equipment outside the academic world. These MRI units are still completely adequate for most MRI examinations acquired for both diagnostic and research purposes. In addition, we used a 32-channel phased array coil, which enabled a very high signal-to-noise ratio. Similarly, we have calculated rADC values considering a normal-appearing region on T2w and contrast-enhanced T1w sequences. The manual placement of ROIs could be considered another limit of our study, even if it was performed by a neuroradiologist with more than fifteen years of experience in the field of glioma MR imaging. Furthermore, a statistical correlation between the OS of the included patients and any comorbid conditions is lacking. However, as mentioned above, the IDH-Mut patients were basically younger than the IDH-Wt ones. This could be in favor of longer survival, regardless of the mutational status.

Lastly, the simplified description of the diffusion process assumed in DWI sequences does not permit to completely map the complexity underlying cellular components and structures, which hinder and restrict the diffusion of water molecules. Thus, the use of more advanced MRI pulse sequences and a higher order of diffusion model (for example, through the use of multiple b values for diffusion kurtosis imaging analyses) may partially overcome these limits at the cost of less user-friendly and more time-consuming pre- and post-processing workflows.

## 5. Conclusions

In our study, an MRI demonstrated an optimal diagnostic performance for imaging prediction of IDH mutations. Moreover, DWI is routinely performed in every MRI protocol in the assessment of brain tumors, and our results corroborate the utility of this simple imaging biomarker for radiogenomic correlation. Adding quantitative data such as the evaluation of ADC values to cMRI could be used routinely as an easy noninvasive marker of specific molecular patterns and could help decision-making process in patients with glioma. Moreover, imaging predictions of IDH mutations will become valued as IDH-mutant inhibitors develop clinically for neoadjuvant therapy [36]. Lastly, prospective studies with larger patient samples such as multicenter studies with different MRI equipment are needed to confirm these results and to validate this method.

## Figures and Tables

**Table 1:** The main clinical and conventional MRI (cMRI) features of the isocitrate dehydrogenase (IDH) mutational status in grade III astrocytomas.

	IDH Mutation (10)	IDH Wild-Type (12)	*p*-Value
Sex (male/female)	6/4	8/4	0.564
Age	43 ± 13	58 ± 14	* 0.041
Location			0.456
Frontal lobe	8	6	
Parietal lobe	0	2	
Temporal lobe	2	3	
Occipital lobe	0	0	
Insular lobe	0	1	
T2w Signal Intensity			0.092
Homogeneous	3	8	
Heterogeneous	7	4	
Borders			0.194
Defined	7	4	
Indistinct	3	8	
Edema			0.665
Yes	5	8	
No	5	4	
Contrast enhancement			* 0.039
Yes	0	5	
No	10	7	

* *p*-value < 0.05.

**Table 2:** Comparison of mean apparent diffusion coefficient (ADCm) and relative ADC (rADCm) values between IDH-mutant (Mut) and wild type (Wt) grade III astrocytomas. IDH-Mut tumors show significantly higher ADCm and rADCm values compared with IDH-Wt. Higher ADCm values in IDH-Mut grade III astrocytomas were confirmed also after statistical correction for location and age (*).

	IDH-Mutation	IDH Wild Type	*p*-Value
ADCm	1.21 ± 0.19	0.90 ± 0.08	0.0004 (* 0.0012)
rADCm	1.77 ± 0.33	1.33 ± 0.13	0.0021
